# Biomechanical Properties of Blood Plasma Extracellular Vesicles Revealed by Atomic Force Microscopy

**DOI:** 10.3390/biology10010004

**Published:** 2020-12-23

**Authors:** Viktor Bairamukov, Anton Bukatin, Sergey Landa, Vladimir Burdakov, Tatiana Shtam, Irina Chelnokova, Natalia Fedorova, Michael Filatov, Maria Starodubtseva

**Affiliations:** 1 Petersburg Nuclear Physics Institute Named by B.P. Konstantinov of NRC «Kurchatov Institute», 1, Orlova Roshcha, 188300 Gatchina, Russia; landa_sb@pnpi.nrcki.ru (S.L.); burdakov_vs@pnpi.nrcki.ru (V.B.); shtam_ta@pnpi.nrcki.ru (T.S.); fedorova_nd@pnpi.nrcki.ru (N.F.); filatov_mv@pnpi.nrcki.ru (M.F.); 2 Alferov Saint Petersburg National Research Academic University of the Russian Academy of Sciences, 8/3, Khlopina St., 194021 Saint Petersburg, Russia; antbuk.fiztek@gmail.com; 3 Institute for Analytical Instrumentation of the Russian Academy of Sciences, 31-33A, Ivana Chernych, 198095 Saint Petersburg, Russia; 4 National Research Center “Kurchatov Institute”, 1, Akademika Kurchatova pl., 123182 Moscow, Russia; 5 Institute of Radiobiology of NAS of Belarus, 4, Fedyuninskogo St., 246007 Gomel, Belarus; irenachelnokova@gmail.com (I.C.); marysta@mail.ru (M.S.); 6 Department of Medical and Biological Physics, Gomel State Medical University, 5, Lange St., 246000 Gomel, Belarus

**Keywords:** extracellular vesicles, exosomes, exomeres, atomic-force microscopy, quantitative nanomechanical mapping

## Abstract

**Simple Summary:**

Exosomes are nanoscale membrane extracellular vesicles that are involved in intercellular communication and signaling, and are a promising tool in biomedicine for drug delivery. Despite the progress in practical application and morphological characterization, information about their biomechanical properties is still scarce. The presence of non-membrane particles called exomeres with similar functions has recently been reported. We applied the atomic force microscopy technique to study the biomechanical properties of both types of particles in air and in liquid. We found a correlation between the biomechanical properties of the vesicles, their size, structure, and function. Our data provide useful information for a better understanding of the biomechanical characteristics of extracellular vesicles and non-membrane extracellular particles and their AFM detection.

**Abstract:**

While extracellular vesicles (EVs) are extensively studied by various practical applications in biomedicine, there is still little information on their biomechanical properties due to their nanoscale size. We identified isolated blood plasma vesicles that carried on biomarkers associated with exosomes and exomeres and applied atomic force microscopy (AFM) to study them at single particle level in air and in liquid. Air measurements of exosomes revealed a mechanically indented internal cavity in which highly adhesive sites were located. In contrast, the highly adhesive sites of exomeres were located at the periphery and the observed diameter of the particles was ~35 nm. In liquid, the reversible deformation of the internal cavity of exosomes was observed and a slightly deformed lipid bi-layer was identified. In contrast, exomeres were not deformed and their observed diameter was ~16 nm. The difference in diameters might be associated with a higher sorption of water film in air. The parameters we revealed correlated with the well-known structure and function for exosomes and were observed for exomeres for the first time. Our data provide a new insight into the biomechanical properties of nanoparticles and positioned AFM as an exclusive source of in situ information about their biophysical characteristics.

## 1. Introduction

Exosomes are a subtype of EVs that are released by the fusion of multivesicular bodies with the plasma membrane [1]. Exosomes differ from other types of EVs by a relatively small size (generally accepted 30–150 nm) and the expression of specific exosomal markers (CD9, CD63, CD81, and others) [2]. They were shown to carry cell-specific cargos of proteins, lipids, and genetic materials, thereby participating in intercellular communication [1,2]. As a result of the extensive studies of exosomes over the last decade, their high potential as sources of non-invasive diagnostic biomarkers in various diseases as well as potential carriers of therapeutic agents has been demonstrated [3,4]. The existence of a sub-population of non-membrane particles less than 50 nm called exomeres has recently been shown [5]. Exomeres exhibit limited sets of proteins commonly associated with exosomes, but their specific biomarkers (ApoM, HPS90, and others) have also been identified [5]. Exomeres were considered as non-EVs due to their non-membranous origin [6]. It was shown that exomeres are capable of carrying bioactive cargo [7], and both exosomes and exomeres serve as biomarkers to define cancer [8] or bind the SARS-CoV-2 spike proteins [9].

Despite the fact that proteomic analysis of exosomes and exomeres are well developed [5,10,11] and visualization by means of electron microscopy (EM) techniques [5,12,13,14,15,16,17] or tapping mode AFM [18,19] are well defined, the force spectroscopy measurements of exosomes are still scarcely studied [20,21,22,23,24,25] and for exomeres at the single particle level are lacking. 

In this article, we demonstrated the binding of exosomes and exomeres with their specific markers. We applied quantitative nanomechanical mapping (PeakForce QNM), which is an AFM mode that lets us acquire high-resolution (HR) AFM images with force spectroscopy measurements simultaneously. We resolved the sub-structural features of single particles and revealed biomechanical properties in air and in liquid, which correlated with the structure and function of exosomes and exomeres.

## 2. Materials and Methods

### 2.1. Blood Plasma Samples

Blood plasma samples were obtained from three healthy female volunteers, who gave written informed consent (the average age being 27 years (95% confidence interval of 24–30 years)). The study was approved by the Ethics Committee of Gomel State Medical University. The volunteers had no history of malignancy, immune deficiencies, autoimmune disorders, hepatitis, or HIV infection. The samples were taken using vacuum tubes with ethylenediaminetetraacetic acid (EDTA) buffer. Then, blood was centrifuged at 800× *g* to remove cells. After that, the supernatant was collected and centrifuged at 3000× *g* for 30 min to remove cell debris. The samples were frozen at −80 °C until use.

### 2.2. Extracellular Vesicle Isolation

EVs were isolated by ultracentrifugation according to standard procedure [26]. Briefly, plasma samples were diluted in the buffer solution (20 mM Tris-HCl, 50 mM NaCl, 5 mM EDTA pH 7.2) in the ratio of 1:5 and centrifuged at 20,000× *g* for one hour. After that, the supernatant was collected and ultra-centrifuged for two hours at 110,000× *g*. The precipitate was dissolved in a buffer and then was centrifuged again at 110,000× *g* for three hours. The supernatant was cast and the precipitate was dissolved in 1 mL of a buffer. Isolated EVs were filtered with a 0.22 µm filter (polyethersulfone membrane) and frozen at −30 °C until use.

### 2.3. Nanoparticle Tracking Analysis (NTA)

Size distribution and concentration of EVs were analyzed by a NTA NanoSight LM10 (Malvern Instruments, Malvern, UK) analyzer, equipped with a 405 nm laser (Nano-Sight, Malvern Instruments, Malvern, UK). The suspension of EVs was diluted 10,000 times. Measurements were carried out at 25 °C, the samples were measured four times. Data analysis was performed using NTA 2.3 software.

### 2.4. Dynamic Light Scattering (DLS)

Measurements were performed on a DLS laser correlation spectrometer (INTOX MED LLC, Saint Petersburg, Russia) using a heterogeneous research scheme [27]. The power-spectral density was measured in the band of 16 kHz and 2500 copies were accumulated. The results were presented as a histogram of the particle size distribution *I(R_h_)* where the abscissa was a hydrodynamic radius *(R_h_)*, while the ordinate was a contribution to the total scattering of particles of a given size *I* % (see Supplementary Materials). In this case, the total scattering of all particles of the sample was taken as 100%. Statistical processing of the obtained data was done using the regularization algorithm [27] with QELS 3.2 software, Gatchina, Russia.

### 2.5. Identification of Extracellular Vesicles (EV) Surface Biomarkers

To determine the presence of specific surface markers on particles of various sizes, a combination of immunoprecipitation (IP) and DLS was used as described previously [28]. Briefly, sepharose beads were immobilized with protein A (PrA/S) (Pasteur Institute, Saint Petersburg, Russia). After that, the suspension was diluted 20 times in PBS and incubated with monoclonal antibodies in the ratio of 1:100. The antibodies that are inherent for exosomes as Anti-CD63 (PAB345Hu01), Anti-CD9 (PAB097Hu01), Anti-CD81 (PAB160Hu01), and exomeres as Anti-ApoM (PAC299Hu01) and Anti-HSP90 (PAA823Hu01) were used (Cloud-Clone Corp., Huston, TX, USA). After 10 min of incubation at room temperature, PrA/S with bound antibodies were added to the fraction of isolated EVs in the ratio of 1:10, incubated for 20 min, and then centrifuged at 3000× *g* for 15 min. The supernatant was collected and used for measurements by DLS (five times for each sample). In control samples, PrA/S and Anti-CD3 (Abcam ab5690, Cambridge, MA, USA) were used.

### 2.6. Atomic Force Microscopy (AFM)

The measurements in air and liquid were carried out using Bioscope Catalyst and Bioscope Resolve microscopes manufactured by Bruker in PeakForce QNM mode. For air measurements, SNL-10 B probes were used. Ten µL of the studied solution diluted in Milli-Q 100 times and was dripped on freshly cleaved mica. After 5 min of incubation, the mica was washed in Milli-Q and left to natural evaporation for four hours. For liquid measurements, ScanAsyst Fluid+ probes were used. The freshly cleaved mica was covered by 10 µL of 0.001 wt% of the poly-L-lysine solution. After 5 min of incubation, the mica was dried in a nitrogen stream, and the 60 µL of the studied solution, previously diluted in Milli-Q 10 times, was dripped. After 5 min of incubation, the probe was wetted in the drop covering the mica surface and the scanning was carried out immediately. Image processing was done using Gwyddion 2.49 software.

## 3. Results

### 3.1. Biomarker Identification Combined with Dynamic Light Scattering (DLS)

**Figure A1:**
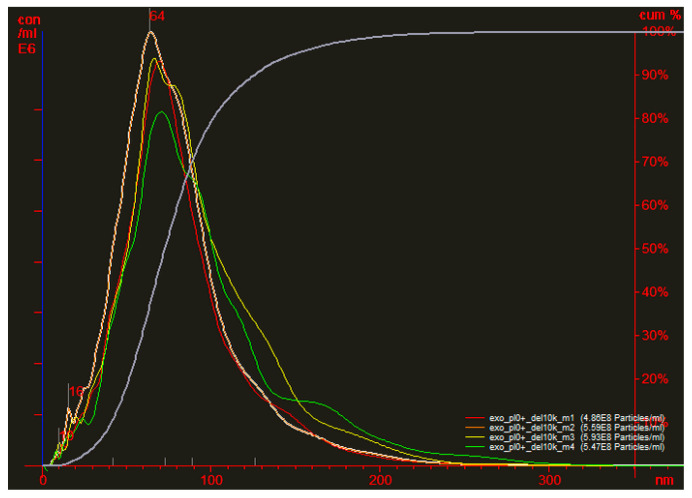
Nanoparticle tracking analysis of particle size and concentration. The initial sample was diluted 10,000 times.

The determined concentration of EVs according to NTA was 5·10^12^ particles/mL (see Figure A1). The samples were diluted as described above and used for DLS measurements. The data of the determined size (corresponding to hydrodynamic diameter) and scattering intensity before and after IP of EVs with specific biomarkers was presented in Table 1. It is necessary to highlight that the particles of large size contribute more to scattering intensity than smaller ones. Therefore, if the contributions to the scattering of large and small particles are equal, the concentration of large particles in the solution is much lower [29]. 

DLS measurements of isolated EVs revealed two average size distributions: 27.8 ± 0.53 and 97.0 ± 1.32 nm. To identify specific exosomal biomarkers, isolated EVs were incubated with Anti-CD9, Anti-CD63, and Anti-CD81 antibodies immobilized on PrA/S. Subsequent mild centrifugation led to the sedimentation of specifically bound EVs from the supernatant. In this case, after Anti-CD9 and Anti-CD63 treatment, the absence of EVs was observed. This fact pointed out the presence of CD9 and CD63 biomarkers for both types of particles in isolated EVs. Note that hereinafter, the third peaks at ~1 μm in diameter and varying intensities appeared. These were caused by contaminations introduced by PrA/S and had no effect on the qualitative analysis. The treatment procedure carried out with Anti-CD81 led to complete sedimentation of particles associated with Peak 1 and partial binding of particles associated with Peak 2. If Anti-CD81 concentration was increased by two times, it did not affect the particles’ binding. Therefore, the partial binding of the particles associated with Peak 2 was observed.

Considering that a set of exomere biomarkers was reported in [5], we used Anti-ApoM and Anti-HSP90 antibodies bound with PrA/S and subsequent treatment procedure. The absence of Peak 1 in the supernatant indicated the specific binding of exomeres in contrast to Peak 2 associated with exosomes. The intensity of Peak 2 expectedly increased, and the scattering intensity was a sum of Peak 2 and Peak 3. In the control samples, the PrA/S and Anti-CD3 were incubated with isolated EVs and then treated, and Peak 1 and Peak 2 were kept. 

### 3.2. The Size, Adhesive Force, and Deformation of EVs Detected by AFM in Air

**Figure 1:**
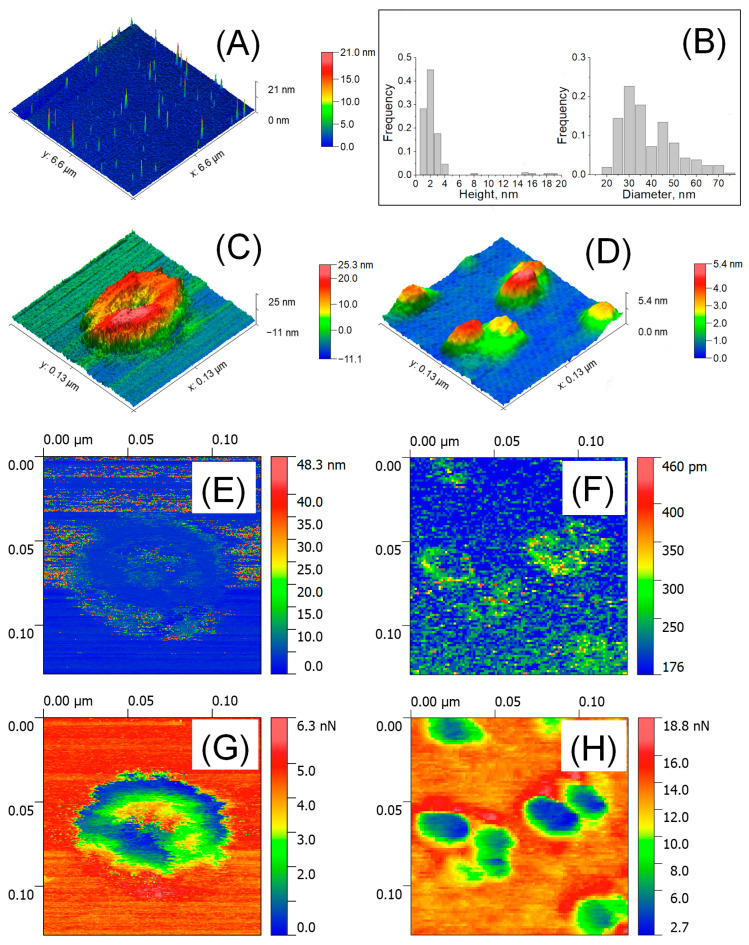
AFM detection of EVs in air. Large scale 6.6 × 6.6 μm^2^ AFM image (**A**) and size distribution (*n* = 264–299) (**B**); HR AFM image 129 × 129 nm^2^ of a single exosome (**C**) and exomeres (**D**); deformation parameters (**E**,**F**): arrows indicated the deformed area; and adhesion forces (**G**,**H**).

Figure 1A shows a large-scale AFM image of isolated EVs in air and their size distribution (Figure 1B). The HR AFM image of a single vesicle associated with exosome is presented in Figure 1C. The cup-shape effect was revealed and was a typical artifact of exosome measurements caused by mechanical indentation by the AFM tip [30]. The observed height was 23.7 nm, while the lateral size calculated as the average of two conjugated diameters was 71.3 nm. Figure 1D shows a near-spherical shape observed for smaller particles that were ~3.16 nm in height and ~31.2 nm in lateral size, which were associated with exomeres previously found in TEM [5] and confirmed our DLS data. The deformation of exosomes was irreversible (Figure 1E). The deformation of exomeres up to ~0.245 nm in height and ~4.23 nm at the periphery was observed (Figure 1F), pointing out the reversible mechanical indentation. Two types of particles differed in the spatial distribution of adhesive forces. Highly adhesive sites of exosome (Figure 1G) were centered at the indented internal cavity (up to 3.45 nN—green color). Adhesive forces of exomeres (Figure 1H) increased from the center (~1.24 nN—blue) to the periphery (up to ~7.6 nN—yellow). The highest adhesive sites (13.8–18.8 nN—red) surrounding the grouped particles were observed.

### 3.3. The Size, Adhesive Force, Deformation and Young’s Modulus of EVs Detected by AFM in Liquid

**Figure 2:**
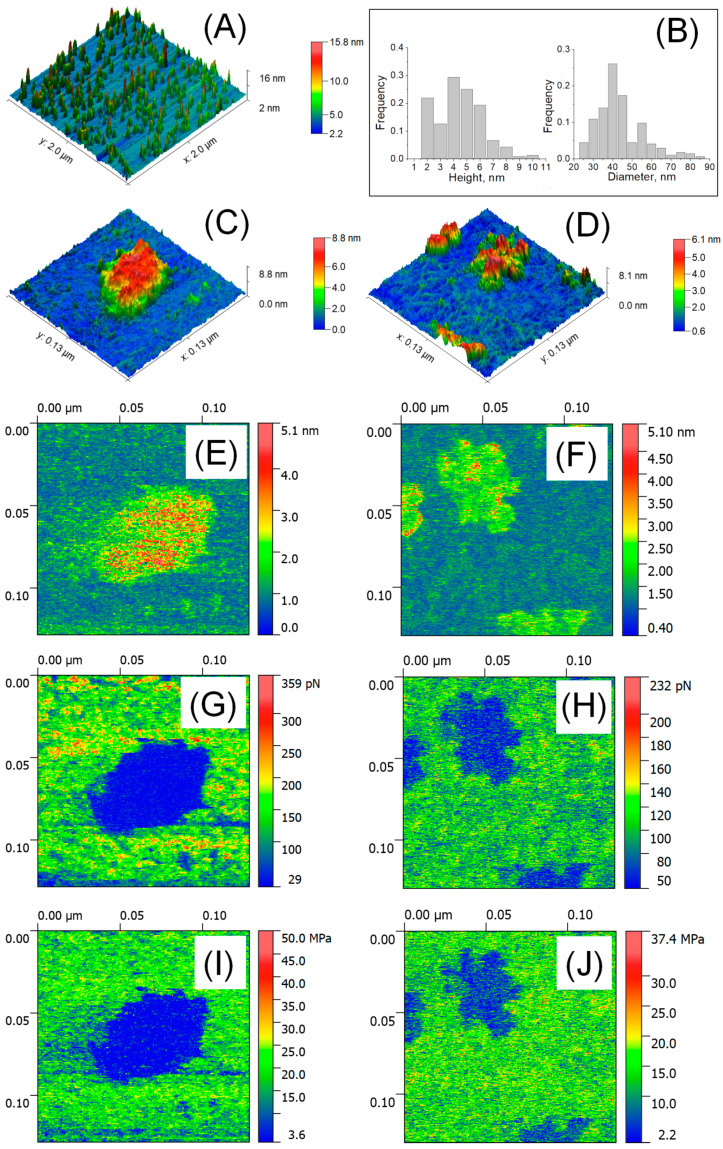
AFM detection of EVs in liquid. Large scale AFM image 2 × 2 μm^2^ (**A**) and size distribution (*n* = 207–254) (**B**); HR AFM image 129 × 129 nm^2^ of a single exosome (**C**), single exomeres and aggregates (**D**); deformation parameters (**E**,**F**), adhesion forces (**G**,**H**), and Young’s modulus (**I**,**J**).

**Figure A2:**
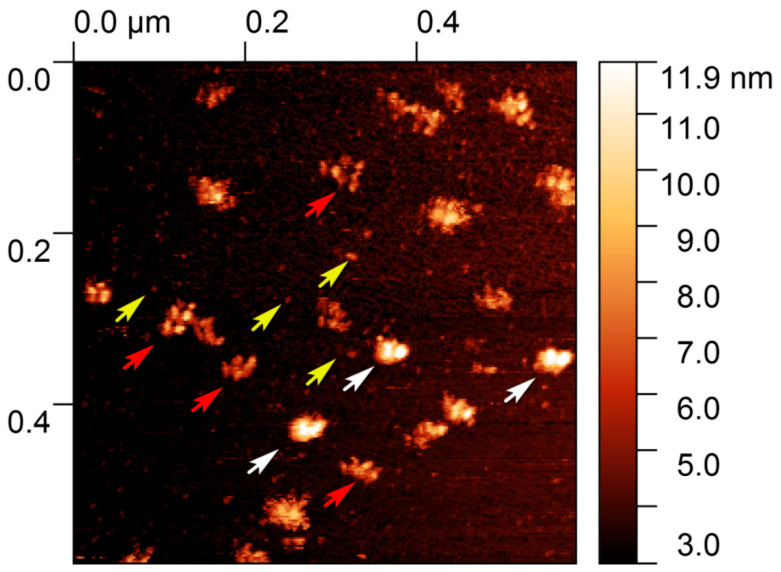
AFM image of isolated EVs in liquid. White arrows indicate exosomes; yellow arrows indicate the single exomeres while red arrows indicate the exomeres, and the nanofilament revealed a “string of beads” morphology. Scan size 600 × 600 nm^2^.

Figure 2A shows a large-scale AFM image of isolated particles in liquid and their size distribution (Figure 2B). The HR AFM image of a single exosome presented in Figure 2C demonstrates a drastic decrease in height up to 6.26 nm, while the lateral size was 70.55 nm. In Figure 2D, the observed height of exomeres was 4.16 nm, with a drastic decrease in lateral size up to 16.3 nm. The formation of aggregates was observed in which particles were intertwined with nanofilaments 1.68–2.2 nm in height showed “string of beads” morphology (see also Figure A2 with scan size 600 × 600 nm^2^). Exosomes as well as exomeres demonstrated reversible deformation. The internal cavity of the exosome (Figure 2E) was deformed up to 4.06 nm (red color) and the slightly deformed rim (green) were resolved. The thickness of the rim was about 6 nm, which corresponded to the thickness of electronically dense lipid bi-layer well defined in Cryo-EM studies [15,31]. To the contrary, exomeres (Figure 2F) were slightly deformed at the center (~0.5–1.3 nm, green), but significant deformation comparable to their height (~3–4 nm, red) was observed at the periphery (commonly 1.5 ± 0.45 nm in width). In the observed aggregate, higher deformed sites might be associated with the particles’ rims. The Young’s modulus of both types of particles was sufficiently low compared to the Young’s modulus of the substrate and was of the order of megapascals: 4.7 MPa for exosome in Figure 2G and 8.8 MPa for exomeres in Figure 2H. The adhesion forces in liquid were negligible and varied between 0.29 nN for exosomes (Figure 2I) and 0.44 nN for exomeres (Figure 2J). No difference in adhesion force and the Young’s modulus for single exomeres and their aggregates was observed.

## 4. Discussion

### 4.1. The Impact of Biomechanical Properties of EVs on the Observed Size

Studying the size and morphology of the EVs by different techniques [32] was accompanied by their own artifacts. Moreover, the measured parameters depend on the sample preparation procedure [16,17,21,25]. DLS allowed us to identify biomarkers associated with exosomes and exomeres, while AFM focused on their biomechanical properties.

In the first thesis, we declared that biomechanical properties play a key role in the observed particles’ morphology. Exosomes in liquid characterized by the low Young’s modulus conditioned their softness and the low height with significant additional deformation. In air, the height changed due to the loss of water of the vesicles’ skeleton. As a result, stiffness was increased and vesicles acquired a near-spherical shape in contrast to a disk-like shape in liquid. Additionally, we observed that one-time irreversible deformation (in air) led to the cup-shape effect associated with the low Young’s modulus (in liquid) in contrast to reversible indentation of stiffer exosomes [30].

The Young’s modulus of exomeres in liquid slightly increased compared with that for exosomes. The low deformation value of exomeres let us deduce a similar height of the particles in air and in liquid. Indeed, air measurements revealed a height decrease of less than 1 nm compared to liquid. The drastic increase in lateral size in air might be caused by AFM detection of water film (Figure 1H—yellow), which was sorbed at adhesive sites of the particles (Figure 1H—green). The observed features were discussed for low-density and high-density lipoproteins elsewhere [33].

### 4.2. Correlation between Biomechanical Properties, Structure, and Function of EVs

The second thesis we proposed is a correlation between biomechanical properties, structure, and function of the studied particles.

As discussed above, the indentation in air as well as the deformation in liquid indicates the presence of a soft internal cavity restricted by a stiffer membrane. The sub-structural features are associated with exosomes, while the absent ones are characteristic of non-membranous exomeres.

Highly adhesive sites of exosomes centered at the indented cavity might be associated with a higher protein density encircled by a membrane. In contrast, highly adhesive sites of exomeres detected in air might be associated with the protein density and serve to bind and carry biocargo [7].

We are attempting to speculate that exomere aggregates bind through tiny nanofilaments. It has been shown that the higher adhesive nanofilaments, revealed on glioblastoma exosomes, might serve as an anchorage of their binding to the cell [23]. In liquid, the aggregates demonstrated no clear difference in the biomechanical properties compared to single exomere particles. In air, the highest adhesive regions might be associated with dried nanofilaments. Due to a lack of knowledge on non-EVs to date, we cannot speculate about the nature of aggregates or the relationship of the observed deformation at the periphery in exomeres in liquid to their function.

## 5. Conclusions

We identified exosomes and exomeres in isolated EV suspension and applied the AFM technique to acquire HR images and force spectroscopy measurements at the single-particle level. Based on the biophysical properties and sub-structural features, we can differentiate exosomes and exomeres in air and in liquid. Based on the biophysical characteristics of exosomes and exomeres detected by our approach, we could predict their morphology after drying. Compared to other techniques, AFM is not only a HR visualization tool, but a valuable source of information about the distribution of the biomechanical properties of the nanosized samples.

## Figures and Tables

**Table 1:** Identification of exosomal and exomeral biomarkers by means of dynamic light scattering (DLS) combined with immunoprecipitation (IP).

Sample	Peak 1		Peak 2		Peak 3	
	*D_h_*, nm	Intensity, %	*D_h_*, nm	Intensity, %	*D_h_*, nm	Intensity, %
Isolated EVs	27.8 ± 0.53	55.1 ± 3.15	97.0 ± 1.32	44.9 ± 3.15	–	–
EVs + PrA/S + AntiCD9	–	–	–	–	750.9 ± 7.23	100 ± 0.00
EVs + PrA/S + Anti-CD63	–	–	–	–	747.2 ± 5.35	100 ± 0.00
EVs + PrA/S + Anti-CD81	–	–	97.2 ± 1.24	39.6 ± 2.50	1343.4 ± 14.20	60.4 ± 2.69
EVs + PrA/S + Anti-ApoM	–	–	94.0 ± 0.81	62.7 ± 4.24	678.9 ± 12.59	37.3 ± 4.24
EVs + PrA/S + Anti-HSP90	–	–	92.4 ± 0.83	61.3 ± 2.96	713.9 ± 9.25	38.7 ± 2.96
EVs + PrA/S	27.9 ± 0.37	55.4 ± 2.77	97.0 ± 1.32	42.6 ± 2.58	727.9 ± 110.48	2.0 ± 0.47
EVs + PrA/S + Anti-CD3	26.34 ± 0.52	63.50 ± 1.70	90.52 ± 0.52	36.36 ± 1.70	1061.14 ± 0.52	0.14 ± 0.04

## Supplementary Materials

The following are available online at https://www.mdpi.com/2079-7737/10/1/4/s1, DLS measurements data.
